# Expression and Genetic Analysis of MicroRNAs Involved in Multiple Sclerosis

**DOI:** 10.3390/ijms14034375

**Published:** 2013-02-25

**Authors:** Elisa Ridolfi, Chiara Fenoglio, Claudia Cantoni, Alberto Calvi, Milena De Riz, Anna Pietroboni, Chiara Villa, Maria Serpente, Rossana Bonsi, Marco Vercellino, Paola Cavalla, Daniela Galimberti, Elio Scarpini

**Affiliations:** 1 Department of Pathophysiology and Transplantation, “Dino Ferrari” Center, University of Milan, IRCCS Ospedale Maggiore Policlinico, Via F.Sforza 35, 20122 Milan, Italy; E-Mails: elisa.ridolfi@unimi.it (E.R.); clodida@gmail.com (C.C.); albicalvi@libero.it (A.C.); milena.deriz@guest.unimi.it (M.D.R.); pb.anna@libero.it (A.P.); chiara.villa1@unimi.it (C.V.); maria.serpente@unimi.it (M.S.); rossana.bonsi@unimi.it (R.B.); daniela.galimberti@unimi.it (D.G.); elio.scarpini@unimi.it (E.S.); 2 Department of Neuroscience, Azienda Ospedaliera Città della Salute e della Scienza di Torino, Corso Bramante 88, 10126 Turin, Italy; E-Mails: vercellino@libero.it (M.V.); pcavalla@cittadellasalute.to.it (P.C.)

**Keywords:** multiple sclerosis, microRNA, gene expression, association analysis

## Abstract

Evidence underlines the importance of microRNAs (miRNAs) in the pathogenesis of multiple sclerosis (MS). Based on the fact that miRNAs are present in human biological fluids, we previously showed that miR-223, miR-23a and miR-15b levels were downregulated in the sera of MS patients *versus* controls. Here, the expression levels of these candidate miRNAs were determined in peripheral blood mononuclear cells (PBMCs) and the serum of MS patients, in addition to three genotyped single nucleotide polymorphisms (SNPs). Mapping in the genomic regions of miR-223, miR-23a and miR-15b genes, 399 cases and 420 controls were tested. Expression levels of miR-223 and miR-23a were altered in PBMCs from MS patients *versus* controls. Conversely, there were no differences in the expression levels of miR-15b. A significantly decreased genotypic frequency of *miR-223* rs1044165 *T/T* genotype was observed in MS patients. Moreover, the allelic frequency of *miR-23a* rs3745453 *C* allele was significantly increased in patients *versus* controls. In contrast, there were no differences in the distribution of miR-15b SNP. In conclusion, our results suggest that miR-223 and miR-23a could play a role in the pathogenesis of MS. Moreover, *miR-223* rs1044165 polymorphism likely acts as a protective factor, while *miR-23a* rs3745453 variant seems to act as a risk factor for MS.

## 1. Introduction

Multiple sclerosis (MS) is the most common autoimmune disease of the central nervous system (CNS) among young adults. The cause of MS is not clear, as the disease develops in genetically susceptible individuals with the contributions of environmental factors, such as infection, sunlight exposure, and vitamin D deficiency [1]. The clinical course of MS is extremely heterogeneous. Different MS subtypes have been described (relapsing–remitting (RR), secondary progressive (SP), and primary progressive (PP)) and, within each subtype, there is also considerable individual variation in disease course [2]. At present, no laboratory measure that reliably correlates with or predicts disease activity and response to therapies exists.

miRNAs are small noncoding RNA molecules that post-transcriptionally regulate gene expression by targeting the 3′ untraslated region (3′ UTR) of specific messenger RNAs (mRNAs). miRNAs play important roles in various biologic processes such as cell proliferation, development, differentiation, metabolism, apoptosis, angiogenesis, inflammation and immunity [3]. In the few past years, aberrant miRNA expression and function were associated with MS [4–11].

Recent evidence showed the presence of stable miRNAs in biological fluids, such as blood, serum, plasma and cerebrospinal fluid (CSF) [12,13]. The origin and the way these circulating miRNAs are secreted are still unknown, but the simplicity of their detection, combined with the low invasive way to obtain them from patients, make circulating miRNAs ideal prognostic biomarkers for monitoring disease course and response to treatment [14].

In our previous study, we showed that expression levels of miR-223, miR-23a and miR-15b were downregulated in the sera of MS patients *versus* healthy controls [15]. Interestingly, target genes of miR-223, miR-23a and miR-15b seem to play a role in MS pathogenesis [15].

The ease with which blood can be obtained in a manner that is minimally invasive to the patient encouraged us to go further in the analyses of miR-223, miR-23a and miR-15b in the cells of this tissue.

In particular, we determined the expression levels of these miRNAs both in PBMCs and sera from MS patients in order to establish a possible correlation between the levels of miR-223, miR-23a and miR-15b inside and outside the blood cells. Moreover, based on the fact that genetic alterations could influence miRNA expression and possibly play a role in disease susceptibility, we genotyped three SNPs, mapping in the genomic regions of miR-223, miR-23a and miR-15b genes.

## 2. Results and Discussion

### 2.1. miR-223 and miR-23a Expression Levels Are Altered in MS Patients *versus* Controls

In the past few years, the identification of miRNAs differently expressed in blood and lesions of MS patients *versus* controls led miRNAs to be considered the new potential prognostic biomarkers for MS [4]. This idea was more reliable with the recent discovery of stable miRNAs in biological fluids, including plasma, serum, urine, saliva and CSF [12,13]. Secreted miRNAs have many requisite features of good biomarkers: stability in biological fluids, sequence conservation across species and easy detection by quantitative PCR [16].

We previously performed an analysis of circulating miRNAs in sera of MS and healthy control subjects, finding a general downregulation of the expression levels of serum miRNAs in MS patients *versus* controls. In particular, miR-223, miR-23a and miR-15b levels were significantly reduced [15]. In the present study, expression levels of miR-223, miR-23a and miR-15b were determined in PBMCs and serum from 15 MS patients and 12 controls (Table 1), as an independent replication. The RRMS patients were in remission phase.

**Figure 1:**
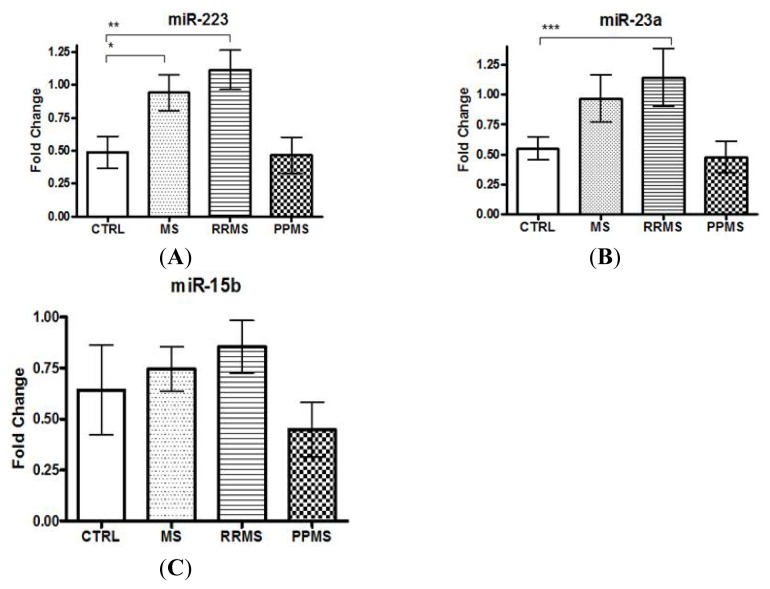
Expression levels of miR-223 (**A**), miR-23a (**B**) and miR-15b (**C**) in PBMCs of MS patients (*n* = 15) and controls (*n* = 12) by Real-time PCR. Mean ± SEM, *p < 0.02; **p = 0.005; ***p < 0.037.

A significantly increased miR-223 relative expression level was observed in PBMCs from MS patients as compared with controls (0.94 ± 0.14 *vs.* 0.49 ± 0.12, *p* < 0.02, Figure 1A). Stratifying according to disease subtype, the upregulation resulted to be even stronger in RRMS patients *versus* controls (1.11 ± 0.15 *vs.* 0.49 ± 0.12, *p* = 0.005) but not in PPMS patients (*p* > 0.050, Figure 1A). Interestingly, miR-223 has already been found upregulated in blood [10,17], and in T regulatory cells [18] from MS compared to healthy subjects and in active MS lesions compared to normal CNS areas in controls subjects [11].

miR-23a levels resulted significantly upregulated only in RRMS patients as compared to controls (1.14 ± 0.24 *vs.* 0.55 ± 0.09, *p* < 0.037, Figure 1B). Conversely, there was no difference in the expression levels of miR-15b between MS patients and controls (*p* > 0.050, Figure 1C).

**Figure 2:**
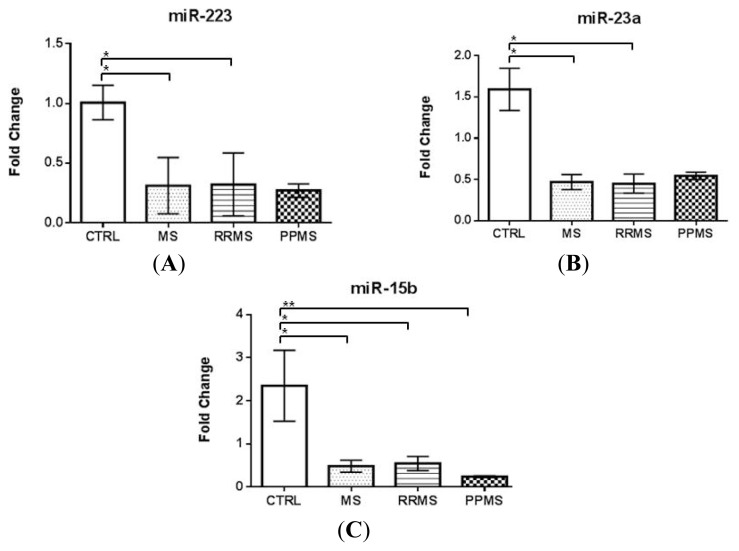
Expression levels of miR-223 (**A**), miR-23a (**B**) and miR-15b (**C**) in serum of MS patients (*n* = 15) and controls (*n* = 12) by Real-time PCR. Mean ± SEM, *p < 0.001; **p < 0.003.

On the contrary, a significant downregulation of miR-223, miR-23a, and miR-15b levels was found in the serum of the same MS population when compared with controls (miR-223: 0.31 ± 0.07 *vs.* 1.00 ± 0.14; miR-23a: 0.47 ± 0.09 *vs.* 1.59 ± 0.26 and miR-15b: 0.48 ± 0.14 *vs.* 2.35 ± 0.82; *p* < 0.001, Figure 2A–C, respectively), in accordance to our previous findings [15]. Moreover, stratifying according to the disease subtype, the downregulation of miR-223 and miR-23a still remained significant in RRMS patients *vs.* controls (*p* < 0.001, Figure 2A,B), whereas miR-15b level was significantly downregulated both in RR- and PPMS patients compared to controls (*p* < 0.003, Figure 2C).

When the correlation analysis was performed, no significant correlations were found between miRNA levels in PBMCs and serum as we expected, and this is probably due to the small number of patients analyzed. Alternatively, the increased expression observed in PBMC could lead to a sequestration of miRNA in such cells, resulting in decreased circulating miRNA levels.

Correlation analysis between miRNA levels in PBMCs or serum and age or sex were carried out, and no statistically significant correlations were found (*p* > 0.05).

The different trend between extracellular and intracellular miRNA levels may reflect a possible role of circulating miRNAs as a novel system of intercellular communication [16]. Circulating miRNAs displayed remarkable stability and resistance to degradation from endogenous RNA activity. The explanation of their exceptional stability possibly resides in their physiological environment such as microvesicles, and exosomes, and in their association to non-vesicle protein [19]. It has recently been shown that exosomes can be secreted by many cells, including T cells, B cells, mast cells, dendritic cells and macrophages [20]. Yang and colleagues demonstrated that functional miRNAs can be transported from macrophages to breast cancer cells, using a co-culture system. Interestingly, exosomes secreted from IL-4 activated macrophages shuttle miR-223 into breast cancer cells and miR-223 promotes breast cancer cell invasion [20]. Based on these findings, a possible explanation of our results could be that the uptake of circulating miR-223 and miR-23a is increased in PBMCs of MS patients, where they exert their function on targeting genes, possibly involved in MS pathogenesis.

Based on TargetScan 6.1, and the www.microRNA.org and www.pictar.org websites, we found predicted target genes of miR-223 and miR-23a involved in immunity and possibly relevant to MS pathology. miR-223 is mainly expressed in myeloid cells and its function was originally described in the regulation of granulopoiesis. It modulates the NF-kb pathway, thus its dysregulation could modulate immune inflammatory responses [21]. miR-23a targets *FGF-2* gene, a member of the fibroblast growth factor family. FGF-2 protein has been implicated in several biological processes, such as limb and nervous system development, wound healing, and tumor growth [22]. FGF-2 levels are reported to be elevated in CSF of MS patients, particular those with active disease [23], and the gene was found to be differentially expressed in active and chronic MS lesions in post-mortem tissues [11], thereby suggesting FGF-2 as a marker of inflammation in MS lesions. Additionally, miR-23a was demonstrated to regulate lamin B1, which is important for myelin maintenance [24].

### 2.2. Genetic Analysis of miR-223, miR-23a and miR-15b in MS Patients

In order to test the genes coding for the studied miRNAs as susceptibility factors for MS, we also decided to perform genetic analyses of miR-223, miR23a and miR-15b. Because of the absence of SNPs in the genes encoding for *miR-223*, *miR-23a* and *miR-15b*, we considered wider genomic regions, corresponding to the linkage disequilibrium (LD) block in which each gene is located. Although the SNPs we identified have not been found in previous Genome Wide Association Study (GWAS), it is not known whether they were included in any of the arrays used. However, our choice was driven by a candidate SNP hypothesis, *i.e*., genetic alterations in candidate miRNA-encoding genes may, to some extent, influence miRNA function, as previously shown in other contexts (see [4] for review).

Allele and genotype frequencies of *miR-223*, *miR-23a* and *miR-15b* SNPs in MS patients and controls were reported in Table 2. A significantly decreased genotypic frequency of *miR-223* rs1044165 *T/T* genotype was observed in MS patients *versus* controls (OR = 0.29, CI: 0.17–0.50; *p* < 0.001). Conversely, a significant increased allelic frequency of *miR-23a* rs3745453 *C* allele in patients with MS as compared with controls was observed (OR = 1.69, CI: 1.28–2.27; *p* < 0.001). No differences in allelic and genotypic distribution of *miR-15b* rs1451761 SNP were observed between patients and controls (*p* > 0.05). No significant correlations were found between these SNPs and miRNA expression levels (*p* > 0.05). The analysis of the three tagging SNPs revealed that the *miR-223* rs1044165T allele likely acts as protective factor, whereas the *miR-23a* rs3745453C allele seems to exert a risk factor for MS pathogenesis. Interestingly, we found that the *miR-223* gene is in LD with the *VISG4* gene, a B7 family-related protein V-set and Ig domain-containing 4. VISG4 is a negative regulator of T cell activation. Vogt *et al.* demonstrated that VISG4 is a strong inhibitor of T cell proliferation *in vitro* and *in vivo*. The restricted expression of this protein on resting tissue macrophages suggests that VISG4 has an important role in the maintenance of T cell unresponsiveness in healthy tissue [25]. Thus, an association with variants in *VISG4* gene in LD with *miR-223* locus would be responsible for the association found with rs1044165 in our MS population. However, a deeper investigation of genetic variability in the *VISG4* gene is required to confirm our hypothesis.

## 3. Experimental Section

This study was approved by the Institutional Review Board (IRB) of the University of Milan, Fondazione Cà Granda, IRCCS Ospedale Maggiore Policlinico. All subjects provided written informed consent.

### 3.1. Subjects

For the genetic analysis, the MS population was recruited at the Multiple Sclerosis Center of Fondazione Cà Granda IRCCS Ospedale Maggiore Policlinico and at the AOU S. Giovanni Battista di Torino. The female-to-male ratio was approximately 2:1 (276 females, 123 males); the mean age was 43.9 ± 0.7 years; the mean age of onset was 32.4 ± 0.6 and the mean disease duration was 12.9 ± 0.6. All patients underwent the standard work up for MS (medical history, physical and neurological examination, screening laboratory tests, brain magnetic resonance imaging (MRI)) and diagnoses were based on the McDonald’s criteria [26]. Four-hundred and twenty healthy volunteers were recruited as controls. The controls were matched for age and geographic origin with the MS patients and had no sign or familial history for neurological diseases.

The characteristics of MS patients and controls enrolled for the expression analysis were summarized in Table 1. All patients considered for miRNA expression level subjects had not received any therapy prior to blood withdrawal. They were newly diagnosed patients that gave their consent for this study prior to starting any immunomodulatory treatment.

### 3.2. DNA Isolation

High-molecular weight DNA was isolated from whole blood using a Flexigene Kit (Qiagen, Hildren, Gemany), as described by the manufacturer. The amount of DNA for each sample was determined by measuring the optical density at 260 nm wavelength using a spectrophotometer (Eppendorf AG, Wesseling-Berzdorf, Germany). DNA samples were aliquoted and stored at −20 °C until used.

### 3.3. Serum Sampling

Whole blood samples were allowed to sit at room temperature for a minimum of 30 min and a max of 2 h, after collection. Separation of the clot was done by centrifugation at 1000–1300× *g* at room temperature for 15–20 min. The serum was removed and dispensed in aliquots of 400 μL into cryo-tubes. Specimens were stored at −80 °C.

### 3.4. miR-223, miR-23a and miR-15b Extraction from PBMCs and Serum and Quantitative Analysis by Real-Time PCR

Total RNA enriched for small RNA species was isolated from PBMCs, using the mirVANA miRNA Isolation Kit according to the instruction of the manufacturer (Applied Biosystems, Grand Island, NY, USA), and the RNA concentration determined by using a NanoDrop ND-3300® Fluorospectrometer.

Four-hundred microliters of human serum was thawed on ice and lysed with equal volume of 2× Denaturing Solution (Ambion, Austin, TX, USA). To allow for sample-to-sample normalization, synthetic *C. elegans* miRNAs *cel-miR*-39 (Qiagen) were added (25 fmol in 5 μL total volume) to each denatured sample [12]. RNA was isolated using mirVANA PARIS kit following the manufacturer’s protocol for liquid samples (Ambion).

The reverse transcription reaction was performed using the TaqMan@MicroRNA Reverse Transcription kit (Applied Biosystems) according to the manufacturer’s instructions. The quantitative Real-time PCR was performed on the ABI 7500 FAST system (Applied Biosystems). miR-223, miR-23a and miR-15b were detected with single TaqMan® miRNA assay (Applied Biosystems). Data were analyzed with SDS Relative Quantification Software version 2.2.2 (ABI: Foster, CA, USA), with the automatic Ct setting for assigning baseline and threshold for Ct determination. Relative expression levels were calculated as Fold-Change ((−Delta Delta Ct)^2^), which was the normalized gene expression ((−Delta Ct)^2^) in the MS group divided by the normalized gene expression ((−Delta Ct)^2^) in the control group, as previously described [9,27]. The *p* value cut off by *t*-test is *p* < 0.05.

### 3.5. Genotyping

We used Hapmap project (http://hapmap.ncbi.nlm.nih.gov/) and Ensemble browser (http://www.ensembl.org/index.html) to determine the position of miR-223, miR-23a and miR-15b genes and Haploview software (http://www.broad.mit.edu/mpg/haploview/) to identify the tagging SNPs of miR-223, miR-23a and miR-15b genes (rs1044165, rs3745453 and rs1451761, respectively). The SNPs were analyzed using the TaqMan methodology, according to the instructions of the manufacturer.

### 3.6. Statistical Analysis

Statistical analysis was performed using the Sigma Stat 3.1 software (Systat Software Inc, San Josè, CA, USA, 2008). Allelic and genotypic frequencies were obtained by direct counting. Chi square test was used to test for Hardy Weinberg Equilibrium (HWE, http://www.husdyr.kvl.dk/htm/kc/ popgen/genetik/applets/kitest.htm). Both cases and controls were in HWE. Chi Square test was used to test for differences in allele distributions between the groups. Odds ratio (OR) was calculated along with its 95% Confidence Interval (CI).

Expression levels of miR-223, miR-23a and miR-15b were analyzed using the One-Way Anova test.

## 4. Conclusions and Limitations

In this study, we found different expression levels of miR-223 and miR-23a in PBMCs of MS patients compared to controls, suggesting a possible involvement of these two miRNAs in the pathogenic mechanisms of the disease. In particular, we observed a significant upregulation of miR-223 and miR-23a levels in RRMS, but not in PPMS patients, though this could be due to the small number of PPMS cases. Therefore, we are planning to increase the number of PPMS patients in order to confirm our hypothesis.

The genetic analysis showed *miR-223* SNP as a protective factor, and miR-23a variant as a risk factor for MS. However, genetic analysis on a larger population is required to draw definitive conclusions.

## Figures and Tables

**Table 1:** Characteristic of patients and controls in miRNAs expression analysis.

miRNAs expression analysis population	CON	MS-all	RRMS	PPMS
N	12	15	11	4
Gender (M:F)	5:7	4:11	2:9	2:2
Mean age, years ± SEM	33.9 ± 2.4	40.3 ± 3.2	36.2 ± 2.8	51.8 ± 6.7
Mean age at onset, years ± SEM		35.7 ± 2.6	31.4 ± 2.0	45.5 ± 4.6
Mean disease duration, years ± SEM		3.5 ± 1.3	2.1 ± 0.8	6.8 ± 3.5

**Table 2:** Allele and genotype frequencies expressed as *n* (%) of miR-223 rs1044165, miR-23a rs3745453 and miR-15b rs1451761 SNPs in MS patients (*n* = 399) and controls (*n* = 420) by Real-time PCR.

SNP	n°	Genotype *n* (%)			Allele *n* (%)	
*miR-223*						
rs1044165		CC	CT	TT	C	T
Controls	420	348 (82.9)	13 (4.3)	59 (14.0)	709 (84.4)	131 (15.6)
MS patients	399	305 (76.4)	76 (19.0)	18 (4.5) *	686 (86.0)	112 (14.0)

*miR-23a*						
rs3745453						
Controls	420	240 (57.1)	159 (37.9)	21 (5.0)	639 (76.1)	201 (23.9)
MS patients	399	176 (44.1)	168 (42.1)	55 (13.8)	520 (65.2)	278 (34.8) **

*miR-15b*						
rs1451761						
Controls	420	121 (28.8)	201 (47.9)	98 (23.3)	443 (52.7)	397 (47.3)
MS patients	399	117 (29.3)	182 (45.6)	100 (25.1)	416 (49.5)	382 (48.9)

* *p* < 0.001, OR = 0.29, CI: 0.17–0.50;

** *p* < 0.001, OR = 1.69, CI: 1.28–2.27.
